# The Bacterial Poisons

**Published:** 1894-01

**Authors:** 


THE BACTERIAL POISONS. By Dr. N. Gamaleïa. Translated by E. P. Hurd, M. D. Price: paper cover, 25 cents; cloth, 50 cents. George S. Davis, Publisher, Detroit, Michigan.

This is another of the "Physician's Leisure Library Series," and treats of very important subjects, under three heads: First, History of Microbial Toxicology. Second, General Toxicology. Third, Special Toxicology. The book occupies a popular field, and should have a good sale. H.

